# MoRo: From One-Sample Ring-LWE Rounding Key Exchange to Module-LWE IND-CCA KEM

**DOI:** 10.3390/s26123674

**Published:** 2026-06-09

**Authors:** Yuntao Wang, Yuki Otsuka, Tsuyoshi Takagi

**Affiliations:** 1 Graduate School of Informatics and Engineering, The University of Electro-Communications, Tokyo 182-8585, Japan; o2630026@gl.cc.uec.ac.jp; 2 Graduate School of Information Science and Technology, The University of Tokyo, Tokyo 113-8656, Japan; takagi@mist.i.u-tokyo.ac.jp

**Keywords:** Module-LWE, IND-CCA KEM, FO transform, key exchange

## Abstract

With the growing need for long-term secure communications in Internet-of-Things (IoT) and sensor-network environments, practical and robust post-quantum key-establishment mechanisms have become increasingly important. In this work, we revisit the ephemeral-only Ding key exchange (DKE) proposed at ACNS 2019, which is based on one-sample Ring Learning With Errors (Ring-LWE) with rounding, and the original analysis of which covers only passive security. Building on the DKE framework, we propose MoRo-KEM, a Module Learning With Errors (Module-LWE)-based key-encapsulation mechanism using rounding. First, we lift the construction from the Ring-LWE setting to the Module-LWE setting, retaining ring-level efficiency while enabling more flexible parameter choices and reducing reliance on rigid algebraic structure. Second, we replace discrete Gaussian sampling for secrets and errors with centered binomial sampling, thereby simplifying constant-time vectorized implementations while preserving the required noise behavior. Third, we extend the resulting key-exchange core to an IND-CPA-secure public-key encryption scheme and further obtain an IND-CCA-secure KEM via the Fujisaki–Okamoto transform. Finally, at security level I, MoRo-KEM achieves a decryption failure rate of $2^{-166}$, lower than the $2^{-139}$ reported for CRYSTALS-Kyber, thus improving robustness against decryption-failure attacks. These properties make the proposed design attractive for secure key establishment among sensor nodes, edge devices, and gateways operating under constrained computation, memory, and communication budgets. Overall, our construction provides a concrete path from ephemeral key exchange to a practical IND-CCA-secure KEM instantiated over Module-LWE.

## 1. Introduction

### 1.1. Background

IoT and sensor-network environments typically consist of a large number of resource-constrained devices, such as sensor nodes, edge devices, and aggregation gateways, which operate under limited computational power, memory, bandwidth, and energy budgets. At the same time, these systems often require long-term confidentiality and authenticated key establishment, because they are widely deployed in critical application domains, including smart homes, industrial monitoring, environmental sensing, healthcare systems, and intelligent transportation. Such infrastructures may remain in operation for many years and rely on cryptographic algorithms for long-term protection. In 1976, Diffie and Hellman introduced the first practical key-exchange protocol, now known as the Diffie–Hellman key exchange [1]. This seminal work established the feasibility of public-key cryptography and fundamentally influenced the design of many subsequent cryptographic schemes and key-establishment protocols. A representative example is the ElGamal encryption scheme, which can be derived from the Diffie–Hellman framework and whose security relies on the hardness of the discrete logarithm problem. Meanwhile, today, widely deployed public-key cryptosystems include RSA, whose security is based on the presumed hardness of the integer factorization problem.

However, in 1994, Shor showed that both integer factorization and discrete logarithms can be solved in polynomial time on a sufficiently powerful quantum computer [2]. Consequently, if large-scale quantum computers become practical, the security of these conventional schemes would be fundamentally undermined. This threat has motivated the development of post-quantum cryptography (PQC), namely, cryptographic schemes that are believed to remain secure even against adversaries equipped with quantum computers.

Driven by this concern, NIST launched its PQC standardization effort and invited submissions for core public-key primitives, including public-key encryption, key exchange, and digital signatures [3]. As part of the evaluation process, candidates were required to provide both security self-evaluations and performance data. For encryption and key-establishment mechanisms, security was primarily assessed under standard notions such as Indistinguishability under Chosen-Ciphertext Attack (IND-CCA). This process has significantly accelerated the development and analysis of practical post-quantum schemes, especially lattice-based constructions, which have emerged as some of the most promising candidates for standardization and deployment.

Among the main families of lattice-based cryptography, schemes based on the Learning With Errors (LWE) problem are particularly important. The plain LWE setting offers a conservative foundation, but typically leads to relatively large public keys and ciphertexts; a representative example is Frodo [4]. To improve efficiency, algebraically structured variants such as Ring-LWE have been introduced [5], enabling more compact keys and faster polynomial arithmetic. Representative Ring-LWE-based schemes include NewHope [6] and the Ding key exchange (DKE) [7]. A further development is Module-LWE, which provides a middle ground between the stronger structure of Ring-LWE and the more conservative but less efficient plain LWE setting. This design space has proved especially attractive in practice, as exemplified by Kyber, a Module-LWE-based IND-CCA-secure key-encapsulation mechanism (KEM) [8].

Among these schemes, DKE is the most closely related to our work. DKE is a one-sample Ring-LWE-based key-exchange protocol in which the secret and error vectors are ephemeral; that is, freshly sampled for each session and not reused across different executions. This design avoids the long-term reuse of noisy secret material and supports a simple and efficient key-exchange mechanism in the passive security model. To offset the communication overhead introduced by the ephemeral setting, DKE combines Ring-LWE with a rounding-based reconciliation mechanism through the Round function, thereby achieving a practical trade-off between security and efficiency.

From the viewpoint of modern PQC deployment, however, several issues remain open. First, although Kyber achieves excellent overall efficiency, its concrete parameter sets are closely tied to number-theoretic transform (NTT)-friendly design choices, which limits flexibility in parameter selection. Second, the decryption failure rate reported for Kyber512 is larger than $2^{-143}$, and decryption failures have long been recognized as a potential attack surface in lattice-based encryption and KEM constructions. Due to the constraints imposed by NTT-friendly parameters, it remains highly challenging to construct parameter sets that simultaneously achieve both a low decryption failure rate and AES-128-bit security. Therefore, it is meaningful to investigate alternative designs that simultaneously provide greater parameter flexibility and a smaller decryption failure rate. In addition, DKE itself provides only passive security for key-exchange, rather than stronger notions such as IND-CPA security for public-key encryption (PKE) or IND-CCA security for KEMs.

These considerations motivate the present work. We revisit the one-sample Ring-LWE key-exchange with rounding and reconciliation proposed by Ding et al. [7], and extend it in two directions. First, we lift the construction from the Ring-LWE setting to the Module-LWE setting, aiming to retain high efficiency while allowing more flexible parameter choices and reducing reliance on rigid algebraic structure. Second, we show how to transform the resulting rounding-based key-exchange core into a practical IND-CCA KEM through a standard PKE → Fujisaki–Okamoto (FO) transform pipeline. In this way, our work connects an ephemeral one-sample key-exchange paradigm with the security and functionality requirements expected of modern post-quantum KEMs.

Simultaneously, the development of practical post-quantum cryptosystems suitable for IoT and sensor-network settings is an important topic within the scope of post-quantum cryptography for IoT and sensor networks. Recent work has also emphasized the importance and deployment challenges of integrating post-quantum cryptography into networking protocols, connected-device environments (e.g., VANET), and federated machine learning systems [9,10,11,12]. From this perspective, a post-quantum KEM for IoT-oriented deployment should not only provide strong security guarantees, but also support efficient and robust implementation on constrained platforms. In particular, moderate implementation complexity, efficient polynomial arithmetic, simple sampling procedures, and a low decryption failure probability are desirable for practical deployment in large-scale sensor-network systems.

These requirements directly motivate the design of MoRo-KEM. The proposed MoRo-KEM has several features that make it relevant to secure communication in IoT and sensor-network architectures. First, the Module-LWE setting provides a practical balance between security and efficiency, making it suitable for post-quantum key establishment between sensor nodes, edge devices, and gateways. Second, the use of centered binomial distributions instead of discrete Gaussian sampling, simplifies implementation and is more amenable to constant-time and vectorized software or hardware designs, which is beneficial for resource-constrained devices. Third, the reduced decryption failure probability improves the robustness and reliability of repeated key-establishment procedures. This property is particularly important in large-scale sensing systems, where even rare failures may be amplified by frequent protocol executions among many devices. Therefore, beyond its theoretical contribution, MoRo-KEM is motivated by practical security requirements arising from IoT and sensor-network deployments. These features make the proposed construction a relevant post-quantum key-establishment approach for constrained and large-scale networked environments.

Recent studies have further emphasized both the deployment relevance of post-quantum KEMs in IoT environments [13,14] and the need for careful treatment of FO-based decapsulation and decryption-failure behavior in post-quantum KEMs [15,16,17]. Moreover, the 2025 NIST fourth-round status report highlights the continuing standardization and diversification of post-quantum key-establishment mechanisms [18].

### 1.2. Our Contributions

In this paper, we revisit the ephemeral-only Ding key exchange (DKE) [7], a one-sample key-exchange protocol based on Ring-LWE, the original analysis of which is restricted to the passive security model. Building on DKE, we propose MoRo-KEM and make the following contributions.

1. **Lifting to the Module-LWE setting**: We lift the original Ring-LWE-based construction to the Module-LWE setting. This preserves the implementation efficiency of ring-based instantiations while enabling more flexible choices of module rank and parameters, and it reduces reliance on rigid algebraic structure, thereby improving robustness against structure-exploiting attacks.
2. **Replacing discrete Gaussian sampling with centered binomial sampling**: We sample the secret vector s and error vector e from a centered binomial distribution (CBD) instead of a discrete Gaussian distribution. This preserves the required noise behavior while enabling simpler constant-time and vectorized implementations.
3. **From key exchange to public-key encryption and IND-CCA KEM**: Following the DKE paradigm, we extend the key-exchange core to an IND-CPA-secure PKE scheme. We then apply the Fujisaki–Okamoto transform [19] to obtain an IND-CCA-secure KEM.
4. **Reducing the decryption failure rate**: The decryption failure rate of CRYSTALS-Kyber is $2^{-139}$, which is larger than $2^{-143}$, motivating concern about attacks exploiting decryption errors, such as decryption-failure attacks. In contrast, MoRo-KEM with q=7681 achieves a decryption failure rate of $2^{-166}$, which is significantly lower and thus improves robustness against such attacks.
  Note that the second-round submission of Kyber estimated the decryption failure rate of Kyber512 to be approximately $2^{-160}$, but this estimate was later considered overly conservative and was revised to approximately $2^{-139}$ in the third-round submission.

These properties also make MoRo-KEM relevant to post-quantum key establishment in IoT and sensor-network scenarios, where lightweight implementation and communication robustness are both important.

### 1.3. Organization

The rest of this paper is organized as follows. Section 2 introduces the necessary preliminaries. Section 3 introduces cryptographic constructions related to our work, including CRYSTALS-Kyber and the Ding key exchange. Section 4 presents the proposed scheme and proves its correctness. Section 5 provides the security analysis. Section 6 presents concrete parameter sets for the proposed scheme. Finally, Section 7 concludes the paper.

## 2. Preliminaries

### 2.1. Notation

We first fix the notation and symbols used throughout this paper. Let *p* and *q* be moduli with p<q, and let *k* denote the module rank. We assume that *n* is a power of two. Let η denote the parameter of the centered binomial distribution. We write Z for the set of integers and $Z_{q}=Z/qZ$ for the ring of integers modulo *q*. We define the polynomial ring $R=Z\left[x\right]/\left(x^{n}+1\right)$ and its reduction modulo *q* by $R_{q}=Z_{q}\left[x\right]/\left(x^{n}+1\right)$. We also define $\beta_{p}=\left\lceil \log_{2}p\right\rceil$ and $\beta_{q}=\left\lceil \log_{2}q\right\rceil$ as the bit lengths used for compression. Let $\left|\right|\cdot {\left|\right|}_{\infty }$ be the $l_{\infty }$-norm. In particular, for a vector x with polynomial components, we denote its infinity norm by ${∥x∥}_{\infty }$, which is the maximum absolute value of the polynomial coefficients. We write $x\overset{\$}{\leftarrow }\chi$ to denote sampling from a distribution χ. We use ⌊x⌋, ⌈x⌉, and ⌊x⌉ to denote the floor, ceiling, and rounding functions, respectively. Concatenation is denoted by a‖b, and a[i] denotes the *i*-th element of an array *a*.

In this paper, elements of $Z_{q}$ are represented by their centered representatives in $\left\{-\frac{q-1}{2},\ldots ,\frac{q-1}{2}\right\}$ rather than (0,q−1). Vectors are denoted by bold lowercase letters, for example, a, and matrices by bold uppercase letters, for example, A.

### 2.2. Probability Distributions

**Centered binomial distribution (CBD)**: For η∈N, the centered binomial distribution ${\mathrm{CBD}}_{\eta }$ is defined as the distribution of the random variable $\sum_{i=1}^{\eta }\left(a_{i}-b_{i}\right)$, where $a_{i}$ and $b_{i}$ are independent and uniformly sampled from {0,1}. Its support is the set of integers in the interval [−η,η].
**Uniform distribution**: The uniform distribution over a finite set *S* is denoted by U(S) and is defined as the distribution of a random variable *X* that takes each value in *S* with equal probability. Formally, if $S=\{s_{1},s_{2},\ldots ,s_{n}\}$, then
  $$X∼U\left(S\right)\;⟹\;\mathrm{Pr}\left[X=s_{i}\right]=\frac{1}{n},\;\forall i=1,\ldots ,n.$$
  Let U[a,b] denote the uniform distribution over the discrete set {a,a+1,…,b}. A random sample from this distribution is written as $X\overset{\$}{\leftarrow }U\left[a,b\right]$. For example, the uniform distribution over the set {0,1,…,q−1} used in this paper is written as $X\overset{\$}{\leftarrow }U\left[0,q-1\right]$.

### 2.3. Symmetric Primitives

Let B denote the set of byte values. We write $B^{\ell }$ for the set of *ℓ*-byte strings and B* for the set of byte strings of arbitrary length. Our IND-CCA KEM construction relies on the following symmetric primitives. We use a pseudorandom function (PRF),

$$\mathrm{PRF}:B^{32}\times B\to B*,$$

and an extendable-output function (XOF),

$$\mathrm{XOF}:B^{*}\times B*\times B*\to B*.$$

We also use two hash functions,

$$H:B*\to B^{32},\;G:B*\to B^{32}\times B^{32},$$

and a key-derivation function (KDF),

KDF:B*→B*.

Furthermore, for a∈B* and c∈Z, we write a+c to denote the subarray of *a* starting at the *c*-th byte.

### 2.4. Lattice Problems

**Learning With Errors (LWE) problem [20]**: Let $A\in Z_{q}^{m\times n}$ be sampled uniformly at random, where $m,n\in Z_{>0}$. Let s be a small secret vector and e be an error vector, sampled from an appropriate noise distribution, for example, $D_{\sigma }$. Then, the pair
  $$\left(A,b=As+e\;\mathrm{mod}\;q\right)\in Z_{q}^{m\times n}\times Z_{q}^{m}$$
  is called an LWE instance. Search version of the LWE problem: Given an LWE instance (A,b), recover the pair (s,e). Decision version of the LWE problem: Given (A,b), distinguish whether b is generated from an LWE instance or is sampled uniformly at random from $Z_{q}^{m}$.

**Ring-LWE problem [5]**: Let m≥1 be a power of 2, let q≥2 be an integer, and let
  $$R_{q}=Z_{q}\left[x\right]/\Phi_{m}\left(x\right),$$
  where $\Phi_{m}\left(x\right)=x^{n}+1$ is the *m*-th cyclotomic polynomial with n=m/2. For a secret polynomial $s\overset{\$}{\leftarrow }\chi$ and an error polynomial $e\overset{\$}{\leftarrow }\chi$, choose $a\in R_{q}$ uniformly at random and output
  $$\left(a,b=a\cdot s+e\right)\in R_{q}\times R_{q}.$$
  Search version of the Ring-LWE problem: Given polynomially many samples $\left(a,b=a\cdot s+e\right)\in R_{q}\times R_{q}$, recover the pair (s,e). *Decision version of the Ring-LWE problem*: Given $a\in R_{q}$, distinguish whether $b=a\cdot s+e\in R_{q}$ is generated from a Ring-LWE instance or is sampled uniformly at random from $R_{q}$.

**Module-LWE problem [21]**: Let $R_{q}=Z_{q}\left[x\right]/\left(x^{n}+1\right)$, and fix integers k,m∈N. Sample $A\leftarrow U(R_{q}^{m\times k})$, $s\leftarrow \chi^{k}$, and $e\leftarrow \chi^{m}$ independently, where χ is applied coefficient-wise to elements of $R_{q}$. Then, the pair
  $$\left(A,\;As+e\right)\in R_{q}^{m\times k}\times R_{q}^{m}$$
  is called a Module-LWE instance. Search version of the Module-LWE problem: Given multiple samples
  $$\left(A,B=As+e\right)\in R_{q}^{m\times k}\times R_{q}^{m},$$
  recover the pair (s,e). Decision version of the Module-LWE problem: Given $A\in R_{q}^{m\times k}$, distinguish whether $B=As+e\in R_{q}^{m}$ is generated from a Module-LWE instance or is sampled uniformly at random from $R_{q}^{m}$.

The Module-LWE assumption, which underlies the security of the key-exchange scheme proposed in this work, states that distinguishing such samples from uniformly random elements is hard for any probabilistic polynomial-time (PPT) adversary.

## 3. Cryptographic Background

In this section, we review prior work on lattice-based key-exchange and encryption schemes. We first introduce the Ding key exchange (DKE) protocol [7], which is an ephemeral Ring-LWE+Rounding-based key-exchange scheme achieving passive security. We then describe several functions from DKE that are essential to our construction. We also briefly review Kyber [8], a Module-LWE-based key-encapsulation mechanism (KEM) that has been standardized by NIST for post-quantum security.

### 3.1. Ding Key Exchange (DKE)

In this subsection, we introduce the key functions and components of DKE [7], which are essential to our proposed construction. In particular, we review the Hint Function, Signal Function, Reconciliation Function, Rounding Function, Recovering Function, and the derivation of a.

**Hint Function**: The Hint Function indicates whether a value lies in a specified region and is defined as follows:
  $$\sigma_{0}\left(x\right)=\left\{\begin{array}{cc} 0, & x\in \left[-\left\lfloor \frac{q}{4}\right\rfloor ,\left\lfloor \frac{q}{4}\right\rfloor \right],\\ 1, & \mathrm{otherwise}, \end{array}\right.$$
  and
  $$\sigma_{1}\left(x\right)=\left\{\begin{array}{cc} 0, & x\in \left[-\left\lfloor \frac{q}{4}\right\rfloor +1,\left\lfloor \frac{q}{4}\right\rfloor +1\right],\\ 1, & \mathrm{otherwise}. \end{array}\right.$$
  Here, $x\in Z_{q}$, and $\sigma_{0}\left(x\right),\sigma_{1}\left(x\right)\in \left\{0,1\right\}$.

**Signal Function**: The Signal Function serves as an indicator used in the reconciliation procedure to determine how nearby values should be grouped. It is defined as follows. For any $y\in Z_{q}$,
  $$\mathrm{Sig}\left(y\right)=\sigma_{b}\left(y\right),$$
  where b←{0,1}. If Sig(y)=1, then *y* is said to lie in the outer region; otherwise, it lies in the inner region. The Signal Function is defined for integers in $Z_{q}$. For $a\in R_{q}$, it is applied coefficient-wise to each coefficient $a_{i}\in Z_{q}$. In this paper, the same notation Sig(·) is used for both the scalar and polynomial versions.

**Reconciliation Function**: The Reconciliation Function is used to enable both parties to derive the same shared value. For any $x\in Z_{q}$, let w=Sig(x). Then,
  $${\mathrm{Mod}}_{2}\left(x,w\right)=\left(\left(x+w\cdot \frac{q-1}{2}\;\mathrm{mod}\;q\right)\;\mathrm{mod}\;2\right).$$
  Here, elements of $Z_{q}$ are first interpreted as integers in Z before the modulo-2 operation is applied.

Let Δ denote the error tolerance of ${\mathrm{Mod}}_{2}\left(\cdot \right)$. Namely, for any $x,y\in Z_{q}$, if ${∥x-y∥}_{\infty }\leq \Delta$, then

$${\mathrm{Mod}}_{2}\left(x,w\right)={\mathrm{Mod}}_{2}\left(y,w\right),$$

where w=Sig(y). In DKE, the value Δ=q/4−2 is used to ensure an overwhelmingly small decryption failure rate.

The Reconciliation Function is defined for integers in $Z_{q}$. For $a\in R_{q}$, it is applied coefficient-wise to each coefficient $a_{i}\in Z_{q}$. In this paper, the same notation ${\mathrm{Mod}}_{2}\left(\cdot \right)$ is used for both the scalar and polynomial versions.

To explain its role, note that the input is first reduced modulo *q* and then modulo 2. Because *q* is odd, the modulo-*q* reduction may change parity only when wrap-around occurs. Such a parity mismatch can occur when one of the two compared values crosses the modulo-*q* boundary while the other does not. The following Signal Function Sig(·) prevents this by adjusting the reconciliation inputs so that both values lie in compatible regions of $Z_{q}$, thereby avoiding asymmetric wrap-around.

**Lemma** **1.** *Let q>8 be an odd integer. The function ${\mathrm{Mod}}_{2}\left(\cdot \right)$ defined above is a robust extractor with respect to the Signal Function Sig(·) with error tolerance*

$$\delta =\frac{q}{4}-2.$$

The proof of Lemma 1 can be found in [22].

**Rounding Function**: The Rounding Function maps an element from $Z_{q}$ to $Z_{p}$. The function Round(x,p,q) is defined in Algorithm 1. Here, we assume that x∈Z and that q>p>0 are integers. The value *x* is a coefficient of a polynomial $a\in R_{q}$, and *q* and *p* are protocol parameters. For convenience, before applying Round(·), we convert
  $$x\in \left\{-\frac{q-1}{2},\ldots ,\frac{q-1}{2}\right\}\;\mathrm{to}\;x\in \left\{0,\ldots ,q-1\right\}.$$
  The Rounding Function is defined for integers in $Z_{q}$. For $a\in R_{q}$, it is applied coefficient-wise to each coefficient $a_{i}\in Z_{q}$. In this paper, the same notation Round(·) is used for both the scalar and polynomial versions.

**Algorithm 1** 
Round(x,p,q)
**Input:**  $x\in Z_{q}$, p,q **Output:**  Rounded value $x^{\prime }$ of *x*
  1:t←⌊2q/p⌋, k←⌊x/t⌋  2:**if** *x* is odd ** then**  3:    $x^{\prime }\leftarrow 2k+1$  4:**else if** *x* is even **then**  5:    $x^{\prime }\leftarrow 2k$  6:**end if**  7:**if** $x^{\prime }=p$ **then**  8:    rnd$\overset{\$}{\leftarrow }U\left[0,1\right]$  9:    **if** rnd=1 **then**10:        $x^{\prime }\leftarrow x^{\prime }-2$11:    **else**12:        $x^{\prime }\leftarrow x^{\prime }+2\;\mathrm{mod}\;\left(p+1\right)$13:    **end if**14:**end if**15:**return** $x^{\prime }$

**Recovering Function**: The Recovering Function is the inverse of the Rounding Function and maps values from $Z_{p}$ back to $Z_{q}$. It is defined in Algorithm 2. Here, we assume that x∈Z and that q>p>0 are integers. The value *x* is a coefficient of a polynomial $a\in R_{q}$, and *q* and *p* are protocol parameters. For convenience, after applying Recover(·), we convert
  $$x\in \left\{0,\ldots ,q-1\right\}\;\mathrm{back}\;\mathrm{to}\;x\in \left\{-\frac{q-1}{2},\ldots ,\frac{q-1}{2}\right\}.$$
  The Recovering Function is defined for integers in Z. For $a\in R_{q}$, it is applied coefficient-wise to each coefficient $a_{i}\in Z_{q}$. In this paper, the same notation Recover(·) is used for both the scalar and polynomial versions.

**Algorithm 2** 
$\mathrm{Recover}(x^{\prime },p,q)$
**Input:** $x^{\prime },p,q$ **Output:**  Recovered value $x^{\prime \prime }$ of $x^{\prime }$
  1:t←⌊q/p⌋  2:**if** $x^{\prime }$ is odd **then**  3:    $x^{\prime \prime }\leftarrow x^{\prime }\cdot t+1$  4:**else if** $x^{\prime }$ is even **then**  5:    $x^{\prime \prime }\leftarrow \left(x^{\prime }+1\right)\cdot t$  6:**end if**  7:**return** $x^{\prime \prime }$



The case distinction in the Round and Recover procedures is introduced to preserve the parity of the reconciliation inputs. Moreover, in the boundary case $x^{\prime }=p$, a random ±2 correction is applied to avoid parity inversion caused by modulo reduction.

**Derivation of polynomial a function**: We use a 128-bit seed to generate a fresh a. Specifically, the seed is given to a pseudorandom number generator, and each coefficient $a_{i}\in Z_{q}$ for i∈[1,n] of $a\in R_{q}$ is derived as in Algorithm 3.

**Algorithm 3** 
${\mathrm{Derive}}_{a}\left(seed\right)$

**Output:** 
Coefficient $a_{i}$ of polynomial $a\in R_{q}$  1:

$a_{i}\overset{\$}{\leftarrow }U\left[0,q-1\right]$





### 3.2. Kyber.CCA-KEM

Kyber [8] constructs an IND-CCA-secure KEM. It first designs an IND-CPA-secure public-key encryption (PKE) scheme and then applies the Fujisaki–Okamoto (FO) transform [19] to obtain an IND-CCA-secure KEM.

More specifically, Kyber follows the standard paradigm of constructing an IND-CCA-secure key-encapsulation mechanism (KEM) by applying the Fujisaki–Okamoto (FO) transform to an underlying IND-CPA-secure public-key encryption (PKE) scheme based on the Module Learning With Errors (Module-LWE) problem. In the context of Kyber, the algorithms Enc and Dec refer to encapsulation and decapsulation, respectively, rather than to encryption and decryption algorithms for a PKE scheme.

Algorithm 4 describes the key-generation procedure, which outputs a public key and a secret key augmented with the auxiliary values required by the FO transform. Algorithm 5 performs encapsulation by sampling a random message, deriving a shared key through a hash-based key-derivation process, and encrypting the message using the underlying IND-CPA-secure PKE. Algorithm 6 performs decapsulation by decrypting the ciphertext, recomputing the expected ciphertext, and outputting the shared key according to the FO verification rule.
**Algorithm 4** Kyber.CCAKEM.KeyGen()**Output:** 
Public key $pk\in B^{12\cdot k\cdot n/8+32}$**Output:** 
Secret key $sk\in B^{24\cdot k\cdot n/8+96}$  1:$z\leftarrow B^{32}$  2:$\left(pk,sk^{\prime }\right):=\mathrm{KyberCPAPKE}.\mathrm{KeyGen}\left(\right)$  3:$sk:=(sk^{\prime }\|pk\|H\left(pk\right)\|z)$  4:**return** (pk,sk)

**Algorithm 5** Kyber.CCAKEM.Enc(pk)
**Input:** 
Public key $pk\in B^{12\cdot k\cdot n/8+32}$**Output:** 
Ciphertext $c\in B^{d_{u}\cdot k\cdot n/8+d_{v}\cdot n/8}$**Output:** 
Shared key K∈B*  1:

$m\leftarrow B^{32}$

  2:

m←H(m)

  3:

$\left(\bar{K},r\right):=G(m||H\left(pk\right))$

  4:

c:=KyberCPAPKE.Enc(pk,m,r)

  5:

$K:=\mathrm{KDF}(\bar{K}||H\left(c\right))$

  6:**return** (c,K)


**Algorithm 6** Kyber.CCAKEM.Dec(c,sk)
**Input:** 
Ciphertext $c\in B^{d_{u}\cdot k\cdot n/8+d_{v}\cdot n/8}$**Input:** 
Secret key $sk\in B^{24k\cdot n/8+96}$**Output:** 
Shared key K∈B*  1:

$pk:=sk+12\cdot k\cdot n/8\in B^{12\cdot k\cdot n/8}$

  2:

$h:=sk+24\cdot k\cdot n/8+32\in B^{32}$

  3:

z:=sk+24·k·n/8+64

  4:

$m^{\prime }:=\mathrm{KyberCPAPKE}.\mathrm{Dec}\left(s,\left(u,v\right)\right)$

  5:

$\left(\bar{K^{\prime }},r^{\prime }\right):=G(m^{\prime }\left||h\right)$

  6:

$c^{\prime }:=\mathrm{KyberCPAPKE}.\mathrm{Enc}\left(pk,m^{\prime },r^{\prime }\right)$

  7:**if** $c^{\prime }=c$ **then**  8:    $K:=\mathrm{KDF}(\bar{K^{\prime }}||H\left(c\right))$  9:
**else**
10:    K:=KDF(z||H(c))11:
**end if**
12:**return** *K*


## 4. Our Proposal

### 4.1. Design Approach

**Figure 1:**
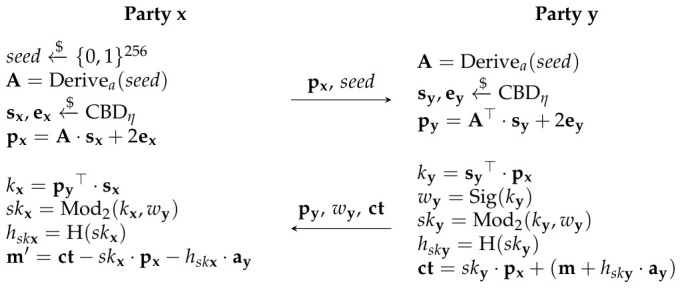
Module-LWE base PKE-protocol

We revisit the one-sample Ring-LWE-based key-exchange scheme and propose a new construction that extends it to the Module-LWE with Rounding setting, which we abbreviate as MoRo. MoRo achieves a simple and secure key-exchange mechanism by leveraging rounding, while benefiting from the efficiency and implementation simplicity of the Module-LWE framework. In this design, the exchanged information is protected by the hardness of the Module-LWE problem, whereas the reconstruction of shared secret values is achieved through a rounding mechanism that suppresses errors. As a result, MoRo maintains high computational efficiency while offering more flexible parameter choices and greater extensibility than existing one-sample Ring-LWE schemes. Furthermore, based on this construction, we design an IND-CPA-secure public-key encryption (PKE) scheme, denoted by MoRo.CPA-PKE, and apply the Fujisaki–Okamoto (FO) transform to obtain an IND-CCA-secure key-encapsulation mechanism (KEM), denoted by MoRo.CCA-KEM. An overview of the proposed construction is shown in Figure 1. Here, $a_{y}$ denotes the k×1 column vector corresponding to the first column of $A^{⊤}$.

### 4.2. MoRo.CPA-PKE

Based on DKE, we construct an IND-CPA-secure public-key encryption (PKE) scheme. Unlike the original DKE setting where the secret values are ephemeral, the proposed PKE uses the secret vector as a static long-term secret key, similar to standard lattice-based PKE constructions such as Kyber. The PKE consists of three algorithms—key generation, encryption, and decryption—which are formally defined in Algorithms 7, 8, and 9, respectively. In Figure 1, Algorithm 7 (MoRo.CPA-PKE: key generation of party x) corresponds to the upper-left part, Algorithm 8 (MoRo.CPA-PKE: encryption of party y) corresponds to the right part, and Algorithm 9 (MoRo.CPA-PKE: decryption of party x) corresponds to the lower-left part.

We define the encoding and decoding procedures as follows. Algorithm 10 gives the pseudocode for the function Decode. This function takes a 32ℓ-byte array $B\in B^{32\ell }$ as input and outputs a polynomial

$$f=f_{0}+f_{1}X+\cdots +f_{255}X^{255},$$

where n=256 and $f_{i}$ belongs to $\left\{0,1,\ldots ,2^{\ell }-1\right\}$. Formally, the function is defined as shown in Algorithm 10. The function Encode (Algorithm 11) is defined as the inverse of Decode.
**Algorithm 7** MoRo.CPAPKE: key generation of party x**Output:** 
Secret key $sk\in B^{\beta_{q}\cdot k\cdot n/8}$**Output:** 
Public key $pk\in B^{\beta_{p}\cdot k\cdot n/8+32}$  1:$d\leftarrow B^{32}$  2:(ρ,σ):=G(d)  3:N:=0  4:**for** i=0 to k−1 **do**  5:    **for** j=0 to k−1 **do**  6:        $\hat{A}\left[i\right]\left[j\right]:=\mathrm{Parse}\left(\mathrm{XOF}\left(\rho ,j,i\right)\right)$  7:    **end for**  8:**end for**  9:**for** i=0 to k−1 **do**10:    $s_{x}\left[i\right]:={\mathrm{CBD}}_{\eta }\left(\mathrm{PRF}\left(\sigma ,N\right)\right)$11:    N:=N+112:**end for**13:**for** i=0 to k−1 **do**14:    $e_{x}\left[i\right]:={\mathrm{CBD}}_{\eta }\left(\mathrm{PRF}\left(\sigma ,N\right)\right)$15:    N:=N+116:**end for**17:$p_{x}:=\hat{A}\cdot s_{x}+2e_{x}$18:$p_{x}^{\prime }=\mathrm{Round}\left(p_{x},p,q\right)$19:$pk:=({\mathrm{Encode}}_{\beta_{p}}\left(p_{x}^{\prime }\;\;\mathrm{mod}\;\;p\right)\|\rho )$20:$sk:={\mathrm{Encode}}_{\beta_{q}}\left(s_{x}\;\;\mathrm{mod}\;\;q\right)$21:**return** (pk,sk)

**Algorithm 8** MoRo.CPAPKE: encryption of party y
**Input:** 
public key $pk\in B^{\beta_{p}\cdot k\cdot n/8+32}$**Input:** 
message $m\in B^{32\cdot k}$**Input:** 
random coin $\sigma^{\prime }\in B^{32}$**Output:** 
ciphertext $ct\in B^{\beta_{p}\cdot k\cdot n/8+n/8+\beta_{q}\cdot k\cdot n/8}$  1:

$\left(p_{x}^{\prime },\rho \right):={\mathrm{Decode}}_{\beta_{p}}\left(pk\right)$

  2:

N:=0

  3:**for** i=0 to k−1 **do**  4:    **for** j=0 to k−1 **do**  5:        ${\hat{A}}^{T}\left[i\right]\left[j\right]:=\mathrm{Parse}\left(\mathrm{XOF}\left(\rho ,i,j\right)\right)$  6:    **end for**  7:
**end for**
  8:**for** i=0 to k−1 **do**  9:    $s_{y}\left[i\right]:={\mathrm{CBD}}_{\eta }\left(\mathrm{PRF}\left(\sigma^{\prime },N\right)\right)$10:    N:=N+111:
**end for**
12:**for** i=0 to k−1 **do**13:    $e_{y}\left[i\right]:={\mathrm{CBD}}_{\eta }\left(\mathrm{PRF}\left(\sigma^{\prime },N\right)\right)$14:    N:=N+115:
**end for**
16:

$p_{y}:={\hat{A}}^{T}\cdot s_{y}+2e_{y}$

17:

$p_{y}^{\prime }=\mathrm{Round}\left(p_{y},p,q\right)$

18:

$p_{x}^{\prime \prime }=\mathrm{Recover}\left(p_{x}^{\prime },p,q\right)$

19:

$k_{y}=s_{y}^{T}\cdot p_{x}^{\prime \prime }$

20:

$w_{y}=\mathrm{Sig}\left(k_{y}\right)$

21:

$sk_{y}={\mathrm{Mod}}_{2}\left(k_{y},w_{y}\right)$

22:

$ct_{1}:=\left({\mathrm{Encode}}_{\beta_{p}}\left(p_{y}^{\prime }\;\;\mathrm{mod}\;\;p\right)\|w_{y}\right)$

23:

$h_{sky}=H\left(sk_{y}\right)$

24:

$ct_{2}:=sk_{y}\cdot p_{x}^{\prime }+\left(m+h_{sky}\cdot a_{y}\right)$

25:

$ct=(ct_{1}\|ct_{2})$

26:**return** ct


**Algorithm 9** MoRo.CPAPKE: decryption of party x
**Input:** 
ciphertext $ct\in B^{\beta_{p}\cdot k\cdot n/8+n/8+\beta_{q}\cdot k\cdot n/8}$**Output:** 
message $m^{\prime }\in B^{32\cdot k}$  1:

$(ct_{1},ct_{2}):=ct$

  2:

$\left(p_{y}^{\prime },w_{y}\right):={\mathrm{Decode}}_{\beta_{p}}\left(ct_{1}\right)$

  3:

$p_{y}^{\prime \prime }=\mathrm{Recover}\left(p_{y}^{\prime },p,q\right)$

  4:

$k_{x}={\left(p_{y}^{\prime \prime }\right)}^{T}\cdot s_{x}$

  5:

$sk_{x}={\mathrm{Mod}}_{2}\left(k_{x},w_{y}\right)$

  6:

$h_{skx}=H\left(sk_{x}\right)$

  7:

$m^{\prime }=ct_{2}-sk_{x}\cdot p_{x}^{\prime }-h_{skx}\cdot a_{y}$

  8:**return** $m^{\prime }$


**Algorithm 10** Decode: $B^{32\ell }\to R_{q}$
  1:**Input:** Byte array $B\in B^{32\ell }$  2:**Output:** Polynomial $f\in R_{q}$  3:

$\left(b_{0},b_{1},\ldots ,b_{256\ell -1}\right):=\mathrm{BytesToBits}\left(B\right)$

  4:**for** i=0 to 255 **do**  5:    $f_{i}:=\sum_{j=0}^{\ell -1}b_{i\ell +j}\cdot 2^{j}$  6:
**end for**
  7:**return** $f_{0}+f_{1}X+f_{2}X^{2}+\cdots +f_{255}X^{255}$


**Algorithm 11** Encode: $R_{q}\to B^{32\ell }$
  1:**Input:** Polynomial $f=f_{0}+f_{1}X+\cdots +f_{255}X^{255}\in R_{q}$, where $f_{i}\in \left\{0,1,\ldots ,2^{\ell }-1\right\}$  2:**Output:** Byte array $B\in B^{32\ell }$  3:**for** i=0 to 255 **do**  4:    **for** j=0 to ℓ−1 **do**  5:        $b_{i\ell +j}:=\left\lfloor \frac{f_{i}}{2^{j}}\right\rfloor \;\mathrm{mod}\;2$  6:    **end for**  7:
**end for**
  8:

$B:=\mathrm{BitsToBytes}(b_{0},b_{1},\ldots ,b_{256\ell -1})$

  9:**return** *B*


### 4.3. Correctness

To prove correctness, we show that if a ciphertext ct is generated as an encryption of a message m, then decrypting ct recovers the same message, that is, $m^{\prime }=m$. Recall that

$$p_{x}=As_{x}+2e_{x},\;p_{y}=A^{⊤}s_{y}+2e_{y}.$$

(1) $$\begin{array}{cc} k_{x}\; & ={\left(p_{y}^{\prime \prime }\right)}^{⊤}s_{x}\\ \; & ={\left(A^{⊤}s_{y}+2e_{y}+d_{y}\right)}^{⊤}s_{x}\\ \; & =s_{y}^{⊤}As_{x}+2e_{y}^{⊤}s_{x}+d_{y}^{⊤}s_{x}, \end{array}$$

and

(2) $$\begin{array}{cc} k_{y}\; & =s_{y}^{⊤}\left(p_{x}^{\prime \prime }\right)\\ \; & =s_{y}^{⊤}\left(As_{x}+2e_{x}+d_{x}\right)\\ \; & =s_{y}^{⊤}As_{x}+2s_{y}^{⊤}e_{x}+s_{y}^{⊤}d_{x}. \end{array}$$

Hence,

$$k_{x}-k_{y}=2e_{y}^{⊤}s_{x}-2s_{y}^{⊤}e_{x}+d_{y}^{⊤}s_{x}-s_{y}^{⊤}d_{x},$$

and therefore

$${∥k_{x}-k_{y}∥}_{\infty }={∥2e_{y}^{⊤}s_{x}-2s_{y}^{⊤}e_{x}+d_{y}^{⊤}s_{x}-s_{y}^{⊤}d_{x}∥}_{\infty }.$$

By Lemma 1, the outputs of ${\mathrm{Mod}}_{2}$ agree whenever the difference is smaller than q/4−2. Accordingly, the decryption failure rate $\delta_{\mathrm{fail}}$ is defined as

(3) $$\begin{array}{c} \delta_{\mathrm{fail}}=\mathrm{Pr}\;\left[{∥2e_{y}^{⊤}s_{x}-2s_{y}^{⊤}e_{x}+d_{y}^{⊤}s_{x}-s_{y}^{⊤}d_{x}∥}_{\infty }\geq \Delta \right]. \end{array}$$

Note that the difference term in (3) consists of linear combinations of the error vectors. Since the error terms in the public values are scaled by a factor of 2, their contribution to the difference is always even. Moreover, the rounding and recovery procedures introduce additional error terms that are also distributed over even values. Therefore, the overall difference between the two reconciliation inputs remains even, and no parity inversion occurs after reduction modulo *q*. Consequently, both parties deterministically derive the same shared bit in {0,1}. The Δ-closeness condition guarantees that the difference between the two inputs remains within the range in which this parity consistency is preserved under the adjustment introduced by Sig(·).

Moreover, the parity-preserving property of the reconciliation procedure follows from the discussion in Section 3.1. Here, the error tolerance is $\Delta =\frac{q}{4}-2$. If this condition is satisfied, then $sk_{x}=sk_{y}$ holds. Moreover, we have

$$ct_{2}=sk_{y}\cdot p_{x}^{\prime }+\left(m+h_{sky}\cdot a_{y}\right).$$

Since $h_{skx}=h_{sky}$, it follows that

$$m^{\prime }=sk_{y}\cdot p_{x}^{\prime }+\left(m+h_{sky}\cdot a_{y}\right)-sk_{x}\cdot p_{x}^{\prime }-h_{skx}\cdot a_{y}=m,$$

which proves correctness.

In this setting, d denotes the error terms introduced by the Round and Recover functions. Let

$$t=\left\lceil \log_{2}q\right\rceil -\left\lceil \log_{2}p\right\rceil ,\;p=\left(p_{1},p_{2},\ldots ,p_{n}\right),\;p^{\prime \prime }=\left(p_{1}^{\prime \prime },p_{2}^{\prime \prime },\ldots ,p_{n}^{\prime \prime }\right).$$

Each element is given by

$$p_{i}^{\prime \prime }=\mathrm{Recover}\left(\mathrm{Round}\left(p_{i},p,q\right),p,q\right),$$

and the error vector is defined as $d=p-p^{\prime \prime }$. Each component $d_{i}$ takes values from

$$\{-2^{t},-2^{t}+2,\ldots ,2^{t}-2\},$$

and these values occur with equal probability, namely,

$$\mathrm{Pr}\left[d_{i}=-2^{t}\right]=\mathrm{Pr}\left[d_{i}=-2^{t}+2\right]=\cdots =\mathrm{Pr}\left[d_{i}=2^{t}-2\right]=\frac{1}{2^{t}}.$$

### 4.4. MoRo.CCA-KEM

By applying the Fujisaki–Okamoto transform to the IND-CPA-secure PKE described above, we construct an IND-CCA-secure key-encapsulation mechanism (KEM). The KEM consists of three algorithms—key generation, encapsulation, and decapsulation—which are formally defined in Algorithms 12–14. In the following, Algorithm 7, Algorithm 8, and Algorithm 9 are denoted by MoRo.CPA-PKE.KeyGen(·), MoRo.CPA-PKE.Enc(·), and MoRo.CPA-PKE.Dec(·), respectively. Although the Fujisaki–Okamoto transform is widely used to achieve IND-CCA security in practical post-quantum KEMs, it also introduces additional implementation considerations, including re-encryption overhead and verification during decapsulation [15,16]. These verification procedures require careful implementation and may introduce additional attack surfaces, including side-channel leakage. Therefore, secure deployment of FO-based constructions requires careful constant-time implementation techniques.
**Algorithm 12** MoRo.CCA-KEM: key generation of party x**Output:** 
secret key $sk\in B^{\beta_{q}\cdot k\cdot n/8+\beta_{p}\cdot k\cdot n/8+32\cdot k+64}$**Output:** 
public key $pk\in B^{\beta_{p}\cdot k\cdot n/8+32}$  1:$z\leftarrow B^{32\cdot k}$  2:$(pk,sk^{\prime }):=\mathrm{MoRo}.\mathrm{CPA}\text{-}\mathrm{PKE}.\mathrm{KeyGen}()$  3:$sk:=(sk^{\prime }||pk||H\left(pk\right)||z)$  4:**return** (pk,sk)

**Algorithm 13** MoRo.CCA-KEM: encapsulation of party y
**Input:** 
public key $pk\in B^{\beta_{p}\cdot k\cdot n/8+32}$**Output:** 
ciphertext $ct\in B^{\beta_{p}\cdot k\cdot n/8+n/8+\beta_{q}\cdot k\cdot n/8}$**Output:** 
shared key K∈B*  1:

$m\leftarrow B^{32\cdot k}$

  2:

m←H(m)

  3:

$\left(\bar{K},\sigma^{\prime }\right):=G(m||H\left(pk\right))$

  4:

$ct:=\mathrm{MoRo}.\mathrm{CPA}\text{-}\mathrm{PKE}.\mathrm{Enc}(pk,m,\sigma^{\prime })$

  5:

$K:=\mathrm{KDF}(\bar{K}||H\left(ct\right))$

  6:**return** (ct,K)


**Algorithm 14** MoRo.CCA-KEM: decapsulation of party x
**Input:** 
ciphertext $ct\in B^{\beta_{p}\cdot k\cdot n/8+n/8+\beta_{q}\cdot k\cdot n/8}$**Input:** 
secret key $sk\in B^{\beta_{q}\cdot k\cdot n/8+\beta_{p}\cdot k\cdot n/8+32\cdot k+64}$**Output:** 
shared key K∈B*  1:

$pk:=sk+\beta_{q}\cdot k\cdot n/8\in B^{\beta_{p}\cdot k\cdot n/8}$

  2:

$H\left(pk\right):=sk+\beta_{q}\cdot k\cdot n/8+\beta_{p}\cdot k\cdot n/8+32\in B^{32}$

  3:

$z:=sk+\beta_{q}\cdot k\cdot n/8+\beta_{p}\cdot k\cdot n/8+64$

  4:

$m^{\prime }:=\mathrm{MoRo}.\mathrm{CPA}\text{-}\mathrm{PKE}.\mathrm{Dec}\left(ct\right)$

  5:

$\left(\bar{K^{\prime }},r^{\prime }\right):=G(m^{\prime }||H\left(pk\right))$

  6:

$ct^{\prime }:=\mathrm{MoRo}.\mathrm{CPA}\text{-}\mathrm{PKE}.\mathrm{Enc}\left(pk,m^{\prime },r^{\prime }\right)$

  7:**if** $ct=ct^{\prime }$ **then**  8:    $K:=\mathrm{KDF}(\bar{K^{\prime }}||H\left(ct\right))$  9:
**else**
10:    K:=KDF(z||H(ct))11:
**end if**



## 5. Security

In this section, we provide the IND-CPA security proof for the proposed public-key encryption (PKE) scheme and the IND-CCA security proof for the resulting key-encapsulation mechanism (KEM) obtained via the Fujisaki–Okamoto (FO) transform. We first introduce several lemmas used in the proofs.

**Lemma** **2.** *In ${\mathrm{Game}}_{2}$, we obtain two Module-LWE samples $\left(A,p_{x}=B_{x}\right)$ and $\left(A,k_{y}=u_{y}\right)$. Assume that the secret and error vectors are sampled from the centered binomial distribution (CBD), denoted by $B_{\eta }$. If $p_{x}$ is a Module-LWE instance, that is,*

$$p_{x}=As_{x}+2e_{x},$$

*then*

$$k_{y}=s_{y}^{⊤}p_{x}=s_{y}^{⊤}As_{x}+2s_{y}^{⊤}e_{x}.$$

*Since the coefficients of $s_{y}$ and $e_{x}$ lie in [−η,η], each coefficient of $s_{y}^{⊤}e_{x}$ is a sum of signed products of independent bounded coefficients. Let $|s_{i}|\leq B_{s}$ and $|e_{i}|\leq B_{e}$. Then, each summand is bounded by $B_{s}B_{e}$. In the case of ${\mathrm{CBD}}_{\eta }$, we may take it that $B_{s}=B_{e}=\eta$, the $B_{s}B_{e}$ follows a discrete distribution with expectation 0, the variance is $\frac{kn\eta^{2}}{4}$ and upper bounded by $\eta^{2}$. Note that the upper bound appears with an extremely small probability of ${\left(2^{-4\eta +1}\right)}^{kn}$, which is interpreted in Appendix A.*

**Lemma** **3.** *Let q>2 be a prime. For a uniformly chosen $a\overset{\$}{\leftarrow }Z_{q}$ and $b\overset{\$}{\leftarrow }\left\{0,1\right\}$, let*

$$w=\mathrm{Sig}\left(a\right)=\sigma_{b}\left(a\right).$$

*Then, the value ${\mathrm{Mod}}_{2}\left(a,w\right)\in \left\{0,1\right\}$ is uniformly distributed.*

**Proof.**  We show that for any c∈{0,1},

$$\mathrm{Pr}\left[{\mathrm{Mod}}_{2}\left(a,w\right)=c|\sigma_{b}\left(a\right)=w\right]=\frac{1}{2},$$

where *a* is uniformly sampled from $Z_{q}$ and *b* is uniformly chosen from {0,1}.

First, consider the interval where $\sigma_{b}\left(a\right)=0$, namely,

R+b:=[⌊−q/4⌋+b,⌊q/4⌋+b].

Its size is

$$\left|R+b\right|=2\left\lfloor \frac{q}{4}\right\rfloor +1.$$

Thus, for uniformly random $a\overset{\$}{\leftarrow }Z_{q}$,

$$\mathrm{Pr}\left[\sigma_{0}\left(a\right)=0\right]=\mathrm{Pr}\left[\sigma_{1}\left(a\right)=0\right]=\frac{2\lfloor q/4\rfloor +1}{q}.$$

Now, define two disjoint subsets of R+b according to the parity of *a*:

$${\left(R+b\right)}_{0}:=\left\{\;a\in R+b|a\equiv 0\;\left(\mathrm{mod}\;2\right)\;\right\},$$

$${\left(R+b\right)}_{1}:=\left\{\;a\in R+b|a\equiv 1\;\left(\mathrm{mod}\;2\right)\;\right\}.$$

Therefore, for c∈{0,1},

$$\mathrm{Pr}\left[{\mathrm{Mod}}_{2}\left(a,0\right)=c\wedge \sigma_{b}\left(a\right)=0\right]=\frac{{|\left(R+b\right)}_{c}|}{q}.$$

Since |R+b|=2⌊q/4⌋+1 is odd, we have the balanced partition

$${|\left(R+0\right)}_{0}{|+|\left(R+1\right)}_{0}{|=|\left(R+0\right)}_{1}{|+|\left(R+1\right)}_{1}|=|R+b|.$$

Hence, conditioning on $\sigma_{b}\left(a\right)=0$, we obtain

$$\begin{array}{cc} & \mathrm{Pr}[{\mathrm{Mod}}_{2}\left(a,0\right)=c|\sigma_{b}\left(a\right)=0]\\ & =\frac{\mathrm{Pr}[{\mathrm{Mod}}_{2}\left(a,0\right)=c\wedge \sigma_{b}\left(a\right)=0]}{\mathrm{Pr}[\sigma_{b}\left(a\right)=0]}\\ & =\left(\frac{1}{2}\cdot \frac{{|\left(R+0\right)}_{c}|}{q}+\frac{1}{2}\cdot \frac{{|\left(R+1\right)}_{c}|}{q}\right)\cdot \frac{q}{2\lfloor q/4\rfloor +1}\\ & =\left(\frac{1}{2}\cdot \frac{\left|R+b\right|}{q}\right)\cdot \frac{q}{2\lfloor q/4\rfloor +1}\\ & =\frac{1}{2}. \end{array}$$

Next, consider the complementary region $Z_{q}\setminus \left(R+b\right)$, where $\sigma_{b}\left(a\right)=1$. Since

$$|Z_{q}\setminus \left(R+b\right)|=q-\left(2\left\lfloor q/4\right\rfloor +1\right),$$

we have

$$\mathrm{Pr}\left[\sigma_{0}\left(a\right)=1\right]=\mathrm{Pr}\left[\sigma_{1}\left(a\right)=1\right]=\frac{q-(2\lfloor q/4\rfloor +1)}{q}.$$

As this region has even size, parity is again uniformly distributed. Thus,

$$\begin{array}{cc} & \mathrm{Pr}[{\mathrm{Mod}}_{2}\left(a,1\right)=c|\sigma_{b}\left(a\right)=1]\\ & =\frac{\mathrm{Pr}[{\mathrm{Mod}}_{2}\left(a,1\right)=c\wedge \sigma_{b}\left(a\right)=1]}{\mathrm{Pr}[\sigma_{b}\left(a\right)=1]}\\ & =\left(\frac{1}{2}\cdot \frac{q-(2\lfloor q/4\rfloor +1)}{q}\right)\cdot \frac{q}{q-(2\lfloor q/4\rfloor +1)}\\ & =\frac{1}{2}. \end{array}$$

Combining the two cases $\sigma_{b}\left(a\right)=0$ and $\sigma_{b}\left(a\right)=1$, we conclude that ${\mathrm{Mod}}_{2}\left(a,w\right)$ is uniformly distributed over {0,1}.    □

**Lemma** **4.** *The vector*

p=As+2e

*can also be regarded as arising from a decisional Module-LWE instance of the form*

b=As+e.

**Proof.**  We have

$$\begin{array}{cc} p=As+2e & \Leftrightarrow 2^{-1}p=2^{-1}As+e\\ & \Leftrightarrow p^{\prime }=A^{\prime }s+e, \end{array}$$

where

$$p^{\prime }=2^{-1}p,\;A^{\prime }=2^{-1}A.$$

Since gcd(q,2)=1, multiplication by $2^{-1}$ in $Z_{q}$ is a bijection. Hence, if A is uniform over $R_{q}^{k\times k}$, then $A^{\prime }$ is also uniform over $R_{q}^{k\times k}$. Therefore, $\left(A^{\prime },p^{\prime }\right)$ can be regarded as a decisional Module-LWE instance.    □

### 5.1. IND-CPA Security

This subsection presents a game-based proof of IND-CPA security for the proposed public-key encryption (PKE) scheme.

**Game**_0_:

This is the real IND-CPA experiment between the challenger and the adversary A. The challenger generates the public parameters honestly and returns the challenge ciphertext corresponding to one of the two challenge messages. Let $S_{i}$ denote the event that A outputs the correct challenge bit in **Game**_*i*_. Then, the IND-CPA advantage of A is

(4) $${\mathrm{Adv}}_{\mathrm{MoRo}.\mathrm{CPAPKE}}^{\mathrm{ind}-\mathrm{cpa}}\left(A\right)=\left|\mathrm{Pr}\left[S_{0}\right]-\frac{1}{2}\right|.$$

**Game**_1_:

This game is identical to **Game**_0_, except that the secret and error vectors are generated from the centered binomial distribution (CBD) using the pseudorandom function (PRF), instead of being viewed as ideally sampled noise.

**Lemma** **5.** *For any PPT adversary A, there exists a PPT adversary $B_{0}$ such that*

$$\left|\mathrm{Pr}\left[S_{0}\right]-\mathrm{Pr}\left[S_{1}\right]\right|\leq {\mathrm{Adv}}_{\mathrm{PRF}}\left(B_{0}\right).$$

**Proof.**  The only difference between **Game**_0_ and **Game**_1_ is whether the randomness used to derive the secret and error vectors is generated by a truly random process or by the PRF. Any distinguisher between these two games therefore yields a distinguisher against the PRF with the same advantage.    □

**Game**_2_:

This game is identical to **Game**_1_, except that A is sampled uniformly at random rather than being deterministically derived from the seed through the extendable-output function (XOF).

**Lemma** **6.** *Assuming that the XOF is modeled as a random oracle, the distributions in* ***Game**_1_ and* ***Game**_2_ are identical. In particular, $\mathrm{Pr}\left[S_{1}\right]=\mathrm{Pr}\left[S_{2}\right]$.*

**Proof.**  In both games, the matrix A is distributed uniformly over the same domain. The only difference is whether it is obtained by explicit uniform sampling or by programming the XOF as a random oracle. Hence, the resulting distributions are identical.    □

**Game**_3_:

This game is identical to **Game**_2_, except that $p_{x}$ is replaced with a uniformly random element $r_{x}$ of the same domain.

**Lemma** **7.** *Assume that the decisional Module-LWE problem is hard. Then, for any PPT adversary A, there exists a PPT adversary $B_{1}$ such that*

$$\left|\mathrm{Pr}\left[S_{2}\right]-\mathrm{Pr}\left[S_{3}\right]\right|\leq {\mathrm{Adv}}_{\mathrm{MLWE}}\left(B_{1}\right).$$

**Proof.**  Given a challenge pair $\left(A,B_{x}\right)$, where $B_{x}$ is either a valid Module-LWE sample or a uniformly random element, the reduction $B_{1}$ embeds $B_{x}$ as $p_{x}$ in the public view given to A. All remaining components are generated honestly. If $B_{x}$ is a valid Module-LWE sample, then the view of A is exactly that of **Game**_2_. If $B_{x}$ is uniform, then the view of A is exactly that of **Game**_3_. Therefore, any distinguisher between **Game**_2_ and **Game**_3_ yields a distinguisher for the decisional Module-LWE problem.    □

**Game**_4_:

This game is identical to **Game**_3_, except that $p_{y}$ is replaced with a uniformly random element $r_{y}$ of the same domain.

**Lemma** **8.** *Assume that the decisional Module-LWE problem is hard. Then, for any PPT adversary A, there exists a PPT adversary $B_{2}$ such that*

$$\left|\mathrm{Pr}\left[S_{3}\right]-\mathrm{Pr}\left[S_{4}\right]\right|\leq {\mathrm{Adv}}_{\mathrm{MLWE}}\left(B_{2}\right).$$

**Proof.**  The proof is analogous to that of Lemma 7. The reduction $B_{2}$ receives a challenge pair $\left(A,B_{y}\right)$ and embeds $B_{y}$ as $p_{y}$. If $B_{y}$ is a valid Module-LWE sample, the view is that of **Game**_3_; if $B_{y}$ is uniform, the view is that of **Game**_4_. Hence, any distinguisher between the two games yields a distinguisher for the decisional Module-LWE problem.    □

**Game**_5_:

This game is identical to **Game**_4_, except that $w_{y}$ is replaced with a uniformly random value of the same length.

**Lemma** **9.** *For any PPT adversary A, $\mathrm{Pr}\left[S_{4}\right]=\mathrm{Pr}\left[S_{5}\right]$.*

**Proof.**  By Lemma 3, the output of the reconciliation bit ${\mathrm{Mod}}_{2}\left(\cdot ,\cdot \right)$ is uniformly distributed once its input is uniform. In **Game**_4_, the value from which $w_{y}$ is derived is already replaced by a uniformly random element. Hence, replacing $w_{y}$ by an explicit uniformly random value does not change the adversary’s view.    □

**Game**_6_:

This game is identical to **Game**_5_, except that the challenge ciphertext is replaced by a uniformly random ciphertext of the same length.

The following lemma isolates the only nontrivial step needed to complete the IND-CPA argument.

**Lemma** **10** (Standard FO-style masking step)**.** *In **Game**_5_, assume that the masking value*

$$sk_{y}\cdot p_{x}^{\prime }+H\left(sk_{y}\right)\cdot a_{y}$$

*is computationally indistinguishable from a uniformly random element of the message space, even given the public view $\left(A,p_{x},p_{y},w_{y}\right)$. Then, for any PPT adversary A, $\left|\mathrm{Pr}\left[S_{5}\right]-\mathrm{Pr}\left[S_{6}\right]\right|\leq \mathrm{negl}\left(\lambda \right)$. This step follows the standard FO-style masking argument; see [23,24,25].*

**Proof.**  In **Game**_5_, the challenge ciphertext has the form

$$ct=\left(ct_{1},ct_{2}\right),\;ct_{2}=sk_{y}\cdot p_{x}^{\prime }+\left(m_{b}+H\left(sk_{y}\right)\cdot a_{y}\right),$$

where *b* is the challenge bit. By the assumption of the lemma, the masking term

$$sk_{y}\cdot p_{x}^{\prime }+H\left(sk_{y}\right)\cdot a_{y}$$

is computationally indistinguishable from a uniformly random element, even conditioned on the public view. Therefore, $ct_{2}$ is computationally indistinguishable from a uniformly random encoding of the same length and, in particular, is independent of the challenge bit *b*. Since $ct_{1}$ is already independent of *b* in **Game**_5_, replacing the entire challenge ciphertext by a uniformly random ciphertext changes the adversary’s success probability by at most a negligible amount.    □

**Theorem** **1.** *Assume that the PRF is secure, the decisional Module-LWE problem is hard, and the hypothesis of Lemma 10 holds. Then, MoRo.CPAPKE is IND-CPA-secure. More precisely, for any PPT adversary A, there exist PPT adversaries $B_{0},B_{1},B_{2}$ such that*

$$\begin{array}{c} {\mathrm{Adv}}_{\mathrm{MoRo}.\mathrm{CPAPKE}}^{\mathrm{ind}-\mathrm{cpa}}\left(A\right)\leq {\mathrm{Adv}}_{\mathrm{PRF}}\left(B_{0}\right)+{\mathrm{Adv}}_{\mathrm{MLWE}}\left(B_{1}\right)+{\mathrm{Adv}}_{\mathrm{MLWE}}\left(B_{2}\right)+\mathrm{negl}\left(\lambda \right). \end{array}$$

**Proof.**  By construction, the challenge ciphertext in **Game**_6_ is independent of the challenge bit. Hence, $\mathrm{Pr}\left[S_{6}\right]=\frac{1}{2}$. Using the triangle inequality together with Lemmas 5–10, we obtain

$$\begin{array}{cc} {\mathrm{Adv}}_{\mathrm{MoRo}.\mathrm{CPAPKE}}^{\mathrm{ind}-\mathrm{cpa}}\left(A\right) & =\left|\mathrm{Pr}\left[S_{0}\right]-\frac{1}{2}\right|\\ & =\left|\mathrm{Pr}\left[S_{0}\right]-\mathrm{Pr}\left[S_{6}\right]\right|\\ & \leq \sum_{i=0}^{5}\left|\mathrm{Pr}\left[S_{i}\right]-\mathrm{Pr}\left[S_{i+1}\right]\right|\\ & \leq {\mathrm{Adv}}_{\mathrm{PRF}}\left(B_{0}\right)+{\mathrm{Adv}}_{\mathrm{MLWE}}\left(B_{1}\right)+{\mathrm{Adv}}_{\mathrm{MLWE}}\left(B_{2}\right)+\mathrm{negl}\left(\lambda \right). \end{array}$$

This proves the theorem.    □

### 5.2. IND-CCA Security (ROM)

We now derive the IND-CCA security of the KEM from the IND-CPA security of the underlying PKE via the Fujisaki–Okamoto (FO) transform. Rather than reproving the FO transform from scratch, we invoke the standard modular ROM analysis of Hofheinz, Hövelmanns, and Kiltz [23].

**Theorem** **2.** *Assume that the hypotheses of Theorem 1 hold and let δ be the decryption-failure probability defined in (3). Then, in the random oracle model (ROM), MoRo.CCAKEM is IND-CCA-secure. More precisely, for any PPT adversary A making at most $q_{RO}$ random-oracle queries, there exists a PPT adversary B such that*

$${\mathrm{Adv}}_{\mathrm{MoRo}.\mathrm{CCAKEM}}^{\mathrm{ind}-\mathrm{cca}}\left(A\right)\leq {\mathrm{Adv}}_{\mathrm{MoRo}.\mathrm{CPAPKE}}^{\mathrm{ind}-\mathrm{cpa}}\left(B\right)+4q_{RO}\delta .$$

*Consequently, by Theorem 1,*

$$\begin{array}{c} {\mathrm{Adv}}_{\mathrm{MoRo}.\mathrm{CCAKEM}}^{\mathrm{ind}-\mathrm{cca}}\left(A\right)\leq {\mathrm{Adv}}_{\mathrm{PRF}}\left(B_{0}\right)+{\mathrm{Adv}}_{\mathrm{MLWE}}\left(B_{1}\right)+{\mathrm{Adv}}_{\mathrm{MLWE}}\left(B_{2}\right)+4q_{RO}\delta +\mathrm{negl}\left(\lambda \right). \end{array}$$

**Proof.**  The claim follows by applying the standard ROM security theorem for the Fujisaki–Okamoto transform [23] to the PKE scheme MoRo.CPAPKE. The required PKE security is provided by Theorem 1, and the only additional loss comes from decryption failures, whose probability is δ. Substituting the bound from Theorem 1 yields the final inequality.    □

### 5.3. IND-CCA Security (QROM)

We next extend the above argument to the quantum random oracle model (QROM). Again, the cleanest formulation is to invoke the standard QROM analysis for the FO transform rather than to sketch the proof directly.

**Theorem** **3.** *Assume that the hypotheses of Theorem 1 hold and let δ be the decryption-failure probability defined in (3). Then, in the quantum random oracle model (QROM), MoRo.CCAKEM is IND-CCA-secure. More precisely, for any quantum adversary A making at most $q_{RO}$ quantum random-oracle queries, there exists an adversary B such that*

$${\mathrm{Adv}}_{\mathrm{MoRo}.\mathrm{CCAKEM}}^{\mathrm{ind}-\mathrm{cca}}\left(A\right)\leq 4q_{RO}\sqrt{{\mathrm{Adv}}_{\mathrm{MoRo}.\mathrm{CPAPKE}}^{\mathrm{ind}-\mathrm{cpa}}\left(B\right)}+8q_{RO}^{2}\delta .$$

*Consequently, by Theorem 1,*

$${\mathrm{Adv}}_{\mathrm{MoRo}.\mathrm{CCAKEM}}^{\mathrm{ind}-\mathrm{cca}}\left(A\right)\leq 4q_{RO}\sqrt{X}+8q_{RO}^{2}\delta ,$$

*where*

$$X={\mathrm{Adv}}_{\mathrm{PRF}}\left(B_{0}\right)+{\mathrm{Adv}}_{\mathrm{MLWE}}\left(B_{1}\right)+{\mathrm{Adv}}_{\mathrm{MLWE}}\left(B_{2}\right)+\mathrm{negl}\left(\lambda \right).$$

**Proof.**  The result follows from the standard QROM security analysis of FO-based KEMs [23,26], applied to the PKE scheme MoRo.CPAPKE. The underlying IND-CPA bound is supplied by Theorem 1, and the additional term is due to the decryption-failure probability δ. Substituting the IND-CPA bound gives the stated inequality.    □

## 6. Parameter Sets

We select the parameters by taking into account the decryption failure rate, computational security, and compatibility with the number-theoretic transform (NTT). The resulting parameter sets are summarized in Table 1, and a comparison with DKE and Kyber is given in Table 2. We propose the parameter set with q=7681 because it provides a larger noise margin and achieves a lower decryption failure rate while maintaining the targeted security level. The decryption failure rate was evaluated using a Python 3.12.3 simulation script based on the error distribution in Equation (3). Compared with the original Ring-LWE-based DKE construction, the transition to the Module-LWE setting increases the public-key and ciphertext sizes due to the module dimension *k* and the associated vector structure. Nevertheless, this trade-off enhances overall robustness by combining the conservative security assumptions underlying LWE with the efficiency gains enabled by NTT-based computation in Ring-LWE. To estimate computational security against the Module-LWE problem, we use the LWE Estimator with the 2016 cost model [27]. More precisely, letting β denote the BKZ block size, the sieving-based attack cost is estimated by

$$T_{\mathrm{sieving}-\mathrm{BKZ}}=8d\cdot 2^{0.292\beta +16.4}\cdot 64\;\left(\mathrm{bits}\right).$$

We report the best-known attack cost in $\log_{2}$ operations and select parameter sets that satisfy NIST Level I security, namely, AES-128-bit security. In particular, our main parameter set achieves a decryption failure rate of $2^{-166}$, which is lower than $2^{-143}$ and therefore provides stronger robustness against decryption-failure attacks.

### Performance of MoRo on Intel Xeon

We evaluated the performance of our MoRo implementation on an Intel Xeon Gold 6240 CPU at 2.60 GHz and report both cycle counts and running time. Our implementation was written in C and compiled with gcc 8.5.0 using the default Makefile settings. For fair comparison, Kyber was also evaluated under the same hardware and software environment. The benchmark output is reported in cycles, which we interpret as CPU-cycle-level cost. For KEM-level evaluation, corresponding to key generation, encapsulation, and decapsulation, we report the median over 10,000 executions, as shown in Table 3. The parameter set used in our experiments is listed in Table 2.

## 7. Conclusions

In this paper, we first constructed a public-key encryption (PKE) scheme based on the DKE mechanism and proved its IND-CPA security. We then obtained a key-encapsulation mechanism (KEM) by applying a slight variant of the Fujisaki–Okamoto (FO) transform to this PKE and proved its IND-CCA security. Moreover, we proposed parameter sets that achieve a decryption failure rate as low as $2^{-166}$ while satisfying AES-128-bit security. In addition, we implemented the proposed scheme and evaluated its performance, demonstrating its practical efficiency.

Beyond the core cryptographic contribution, our results also suggest the relevance of MoRo-KEM to post-quantum protection in IoT and sensor-network environments. In such scenarios, secure key establishment must be achieved under practical constraints on computation, memory, and communication, while maintaining robustness across a large number of distributed devices. The combination of Module-LWE-based efficiency, implementation-friendly sampling, and a low decryption failure rate makes the proposed scheme a meaningful candidate for secure communications between sensor nodes, edge devices, and gateways in long-lived systems that must prepare for the advent of quantum attacks. Accordingly, we believe that the proposed construction provides not only a useful theoretical extension of DKE, but also a practical post-quantum design direction for secure IoT and sensing applications.

## Figures and Tables

**Table 1:** Security parameter candidates for MoRo KEM.

*n*	*k*	*q*	*p*	*t*	η	$\delta_{\mathrm{fail}}$	Estimate for Classical Attacks Using [27]
128	3	7681	1920	2	3	$2^{-181}$	107
256	3	3329	960	2	2	$2^{-44}$	146
256	3	7681	1920	2	2	$2^{-166}$	199

**Table 2:** Comparison of schemes at AES-128 security level.

Scheme	Structure	*n*	*q*	*p*	Security	$\delta_{\mathrm{fail}}$	${\mathrm{Size}}_{\mathrm{pk}}$ (Bytes)	${\mathrm{Size}}_{\mathrm{ct}}$ (Bytes)
Kyber [8]	Module-LWE	256	3329	1024	IND-CCA	$2^{-139}$	800	768
DKE-512 [7]	Ring-LWE	512	120833	7552	Passive	$2^{-60}$	832	1744
MoRo	Module-LWE	256	7681	1920	IND-CCA	$2^{-166}$	1088	2336

**Table 3:** Cycle counts of MoRo for KEM.

Operation	Cycles	Runtime (μs)
Kyber_keypair	88,932	34.094
Kyber_encaps	107,376	41.008
Kyber_decaps	138,336	52.929
MoRo_keypair	162,020	61.989
MoRo_encaps	403,904	154.972
MoRo_decaps	646,702	248.909

## Data Availability

Data is contained within the article. Further inquiries can be directed to the corresponding author.

## Appendix A

In this appendix, we provide the probability corresponding to the upper-bound described in Lemma 2.

For a centered binomial distribution ${\mathrm{CBD}}_{\eta }$, the probability that a coefficient takes the boundary value ±η is

$$\mathrm{Pr}\left[|x|=\eta \right]=2^{-2\eta }.$$

Hence, for two independent coefficients $s_{i},e_{i}∼{\mathrm{CBD}}_{\eta }$, the probability that their product satisfies

$$|s_{i}e_{i}|=\eta^{2}$$

is

$$2{\left(2^{-2\eta }\right)}^{2}=2^{-4\eta +1},$$

where the factor 2 corresponds to the two sign combinations.

Since each coefficient of $s_{y}^{⊤}e_{x}$ contains kn such independent product terms, the probability that all terms simultaneously attain the maximum magnitude $\eta^{2}$ can be estimated as

$${\left(2^{-4\eta +1}\right)}^{kn}.$$
